# Effect of Chitosan Coating Incorporated with *Artemisia fragrans* Essential Oil on Fresh Chicken Meat during Refrigerated Storage

**DOI:** 10.3390/polym13050716

**Published:** 2021-02-26

**Authors:** Milad Yaghoubi, Ali Ayaseh, Kazem Alirezalu, Zabihollah Nemati, Mirian Pateiro, José M. Lorenzo

**Affiliations:** 1 Department of Food Science and Technology, Faculty of Agriculture, University of Tabriz, Tabriz 51666, Iran; m.yaghoubi97@ms.tabrizu.ac.ir (M.Y.); ayaseh@tabrizu.ac.ir (A.A.); 2 Department of Food Science and Technology, Ahar Faculty of Agriculture and Natural Resources, University of Tabriz, Tabriz 51666, Iran; 3 Department of Animal Science, Ahar Faculty of Agriculture and Natural Resources, University of Tabriz, Tabriz 51666, Iran; znemati@tabrizu.ac.ir; 4 Centro Tecnológico de la Carne de Galicia, Parque Tecnológico de Galicia, rúa Galicia n° 4, San Cibrao das Viñas, 32900 Ourense, Spain; mirianpateiro@ceteca.net; 5 Área de Tecnología de los Alimentos, Facultad de Ciencias de Ourense, Universidad de Vigo, 32004 Ourense, Spain

**Keywords:** chitosan, *Artemisia fragrance*, shelf life, chicken fillets, coating

## Abstract

The present study was conducted to assess the impact of chitosan coating (1%) containing *Artemisia fragrans* essential oil (500, 1000, and 1500 ppm) as antioxidant and antimicrobial agent on the quality properties and shelf life of chicken fillets during refrigerated storage. After packaging meat samples, physicochemical, microbiological, and organoleptic attributes were evaluated at 0, 3, 6, 9, and 12 days at 4 °C. The results revealed that applied chitosan (CH) coating in combination with *Artemisia fragrans* essential oils (AFEOs) had no significant (*p* < 0.05) effects on proximate composition among treatments. The results showed that the incorporation of AFEOs into CH coating significantly reduced (*p* < 0.05) pH, thiobarbituric acid reactive substances (TBARS), and total volatile base nitrogen (TVB-N), especially for 1% CH coating + 1500 ppm AFEOs, with values at the end of storage of 5.58, 1.61, and 2.53, respectively. The coated samples also displayed higher phenolic compounds than those obtained by uncoated samples. Coated chicken meat had, significantly (*p* < 0.05), the highest inhibitory effects against microbial growth. The counts of TVC (total viable counts), coliforms, molds, and yeasts were significantly lower (*p* < 0.05) in 1% CH coating + 1500 ppm AFEOs fillets (5.32, 3.87, and 4.27 Log CFU/g, respectively) at day 12. Organoleptic attributes of coated samples also showed the highest overall acceptability scores than uncoated ones. Therefore, the incorporation of AFEOs into CH coating could be effectively used for improving stability and shelf life of chicken fillets during refrigerated storage.

## 1. Introduction

Chicken meat with low amount of lipids and low cost of production not only is a rich source of essential amino acids with high biological value but also is an excellent origin of unsaturated fatty acids and minerals for human body [1]. Its high pH and moisture content make it so that, at aerobic conditions, chicken meat is susceptible to lipid and protein oxidations and microbial growth, leading to a decrease in shelf life [2,3]. Moreover, chicken meat is highly perishable by pathogenic bacteria, such as *Listeria monocytogenes*, *Escherichia coli*, *Campylobacter jejuni*, and *Salmonella* spp. [4]. Today, the major challenge of meat industry is to increase the stability, shelf life, and overall acceptability of the chicken meat by delaying lipid oxidation and preventing microbial growth.

The negative health effects associated with the use of sodium nitrate, benzoic acid, and potassium sorbate as chemical preservatives have recently led researchers and meat industries to focus on applying natural preservatives, such as bacteriocins [5], organic acids [6], essential oils (EOs) [7], or chitosan [2], to delay the lipid and protein oxidations. Plant extracts and EOs from natural origins, generally recognized as safe (GRAS) by the Food and Drug Administration (FDA), have been widely studied [7,8,9,10]. These compounds have been widely used as antimicrobial and antioxidant compounds in food industry due to the presence of terpenoids and phenolic components in their composition [11,12].

Edible coating is a promising technology of active packaging that includes food, packaging, and preservation in a single concept, allowing through the use of biopolymers generated from food industry co-products or underutilized sources of lipids, polysaccharides, or proteins develop an effective system that preserves the quality of food during the shelf life of the product [13]. There is a wide spectrum of natural antioxidants and antimicrobials derived from plants, which have been included as extracts or EOs in films and coatings [14,15]. In this regard, EOs of clove [16], ginger [1], and oregano [17] have been used as antimicrobials in chicken fillets. Chitosan, characterized by high film forming ability, good barrier properties, non-polluting material, biodegradability, non-toxicity, and biocompatibility properties [18,19], and with high antioxidant activity and high antimicrobial effects against wide spectrum of bacteria, yeast, and molds [20,21], has been widely used as antimicrobial coatings and films in meat products. Furthermore, many researchers have been indicated the efficacy of chitosan edible coatings or films to delay quality deterioration and putrefaction in foods [22]. In this regard, high antimicrobial and antioxidant properties of chitosan coatings combined with natural antioxidants have been demonstrated in beef [23], chicken breast meat [24], pork slices [25], and refrigerated pork [26].

The genus Artemisia with more than 500 species belongs to Asteraceae family [27]. *Artemisia fragrans* EO (AFEOs) is not only a rich source of β-thujone, α-thujone, camphor, and 1,8-cineole but also has high antioxidant and antibacterial properties [28,29]. In this regard, techno-functional properties of Artemisia have been indicated in meat and meat products, such as breast and thigh muscles in broilers [30] and Hy-line Brown male chickens [31]. However, there was no reports on chitosan coatings incorporated with AFEOs in chicken meat during storage. Therefore, the aim of this study was to evaluate the preservative effects of this coating on chicken meat during refrigerated storage.

## 2. Materials and Methods

### 2.1. Preparation of Artemisia Fragans Essential Oil (AFEOs)

Clevenger-type apparatus was utilized to production of AFEOs. Dry material of *A. fragrans* (400 g) was immersed in water (1000 mL) and subjected to hydro-distillation. The obtained EO was kept in black glass bottle and stored at 4 °C for further use.

### 2.2. AFEOs Isolation

The gas chromatographic-mass spectrometric (GC–MS) apparatus was used for AFEOs composition (Varian, mod. Saturn 2100T, San Fernando, CA, USA). A fused-silica capillary column (50 m × 0.22 mm, 0.25 µm film thickness) and helium was used as the carrier gas (1 cm^3^/min) were used for compounds separation. Injector and detector temperatures were 280 °C (splitless 20 cm^3^/min) and 260 °C, respectively. Oven condition was 50 °C increased to 250 °C at a rate of 2 °C/min and held for 60 min. The fatty acid methyl ester (FAMEs) were identified by comparison of peaks retention time with standard FAMEs (Sigma-Aldrich, Steinheim, Germany), and the peaks area reported as component percentage [29].

### 2.3. Preparation of Meat Samples

The whole experiment was repeated with a separate source of skinless and boneless chicken breast in five batches during three successive days (5 treatments × 5 time periods × 3 repetitions × 3 runs). The raw material (chicken meats) was bought (weighted between 2.5–5 kg) from a local slaughterhouse and transported directly to laboratory in ice boxes. Ten g chitosan (95% deacetylation degree) was dissolved in 1% acetic acid, reached to 1000 mL. Then, AFEOs was mixed at different concentrations (500, 1000, and 1500 ppm). After that, Tween 80 as a surfactant agent was added to treatment solutions and mixed for 1 min. Based on the previous data on 1% chitosan concentration [32], the chicken breast meats were randomly divided into five groups as follows: T1: Negative control; T2: Treated with distilled water; T3: 1% chitosan (CH) coating + 500 ppm AFEOs; T4: 1% CH coating + 1000 ppm AFEOs; T5: 1% CH coating + 1500 ppm AFEOs. All meat samples, cut with a sterile knife (1 × 3 × 6 cm), were immersed in prepared solutions for 1 h at 4 °C, and, finally, the samples were drained for 2 min and packaged in low density polyethylene bags for evaluation of chemical composition, pH, phenolic compounds, total volatile base nitrogen (TVB-N), thiobarbituric acid reactive substances (TBARS) values, color parameters, organoleptic attributes, and microbial counts at 0, 3, 6, 9, and 12 days of refrigerated storage.

### 2.4. Proximate Composition and pH

Proximate composition of chicken fillet samples, including lipid, ash, protein, and moisture, were determined in triplicate according to Karsli et al. [32]. For evaluation of pH, chicken fillets were homogenized in proportion of 1:10 (*w*/*v*) with distilled water and analyzed with a pH meter (Hanna, Methrom, Switzerland).

### 2.5. Measurement of Thiobarbituric Acid Reactive Substances (TBARS)

The TBARS values of chicken fillets were analyzed according to methodology of Liu et al. [33]. The reactions of thiobarbituric acid with the oxidation products lead to the production of compounds which was measured in a spectrophotometer (Hitachi, Ltd., Tokyo, Japan) at 532 nm. 1,1,3,3-tetraethoxypropane (TEP) was used to prepare the standard curve at concentrations between of 0 to 10 ppm, and the data were expressed as mg malondialdehyde/kg (mg MDA/kg) of chicken meat samples.

### 2.6. Determination of Total Volatile Nitrogen (TVB-N)

Total volatile nitrogen (TVB-N) of meat samples were evaluated by Kjeldahl method with a vapor distillation according to Goulas and Kontominas [34]. The data were reported as mg/100 g of chicken meat samples.

### 2.7. Total Phenolic Content (TPC)

According to Liu et al. [33], total phenolic contents of chicken fillets were evaluated using Folin-Ciocalteu reagent. Firstly, 50 g of chicken meat and 100 mL of boiled distilled water were mixed together and left at room temperature for 20 min. After cooling, the obtained solution was filtered and mixed with Folin–Ciocalteau reagent (2.5 mL) and saturated sodium carbonate solution (5 mL) in test tubes. Finally, the solution was vortexed and held in a dark place (1 h). UV-vis spectrophotometer Hitachi U-3210 (Hitachi, Ltd., Tokyo, Japan) was utilized for the evaluation of TPC at 700 nm. Standard curve was prepared with Gallic acid, and the data was reported as mg/100 g of Gallic acid equivalents (GAE).

### 2.8. Determination of Color Parameters

Color indices (L*: lightness, a*: redness, b*: yellowness) on the surface of the chicken samples were evaluated according to the method proposed by Leon et al. [35] using a simple digital imaging system. The chicken fillets were sized into 1 × 3 × 6 cm thickness to analyze the color. Digital camera with 16 mega-pixels under suitable light at 25 °C and standard plates for instrument calibration were used for capturing the image. Photoshop software was used to analyze the pictures and report the data.

### 2.9. Microbiological Analysis

The microbiological evaluation was performed on days 0, 3, 6, 9, and 12 of the storage period. Twenty-five g of chicken samples were mixed in sterile lab-blender (Neutec, Paddle Lab Blender, Farmingdale, NY, USA) with 225 mL of peptone water (0.1% *w*/*v*; Difco, Becton Dickinson, East Rutherford, NJ, USA) for 3 min. Serial dilutions were prepared with 0.1% peptone water. PCA (Plate Count Agar, Merck, Darmstadt, Germany), VRB (Violet Red Bile Agar, Merck, Darmstadt, Germany) and DRBC (Dichloran Rose-Bengal Chloramphenicol Agar, Merck, Darmstadt, Germany) were employed as nutrient broths for the enumeration of total viable counts (TVC), coliform, mold, and yeast counts, respectively, by pour plate technique. TVC, coliform, mold, and yeast were incubated for 48–72 h at 30 °C, 24 h at 37 °C, and 5 days at 25 °C, respectively. The results were reported as Log10 colony forming unit/g (Log CFU/g) of chicken samples [36].

### 2.10. Sensory Properties

The effects of CH in combination AFEOs on sensory attributes of chicken fillets were evaluated at the end of refrigerated storage. Seventy-two consumers (twenty-four male and forty-eight females) were selected as panelists, all of whom had prior experience about sensory attributes of many kinds of fresh meats. The sensory evaluation consisted of six sessions with twelve panelists for each sitting. A randomized (complete) block design was conducted. The sausage samples were cut into 3-mm thick cubes at room temperature, individually labeled with aleatory numbers and randomly served. Overall acceptability, odor, color, texture, and freshness of chicken fillets were analyzed using hedonic scale (1: really dislike, 5: really like). For increasing accuracy of sensory analysis, between each testing, crackers (unsalted) and water were utilized. Overall acceptability scores were also obtained by average of odor, color, texture, and freshness scores [37].

### 2.11. Statistical Analysis

The experimental data resulted from 5 treatments × 5 time periods × 3 repetitions × 3 runs were analyzed using the statistical software SAS (v.9, SAS Institute Inc., Cary, NC, USA). Normal distribution and variance homogeneity had been previously determined (Shapiro–Wilk). Random block design, considering a mixed linear model, including replicate as a random effect and chicken meat treatment and storage time as fixed effects, were used for the evaluation of pH, TVB-N, and TBARS values, phenolic content, color indexes, sensory characteristics, and microbiological counts. ANOVA (*p* < 0.05), followed by Tukey’s test, was used for moisture, protein, fat, and ash contents. Panelists and sessions were used as random effects for the sensory characteristics. All data were expressed as mean values ± standard error in tables and figures, but the results of chemical properties were expressed as mean values ± standard deviation.

## 3. Results and Discussion

### 3.1. Gas Chromatography-Mass Spectrometry Analysis

The volatile chemical components of AFEOs are shown in Table 1. The data showed that thujone (40.21%) had the highest content and followed by 1,8-Cineole (21.04%), l -camphor (11.87%), and isobornyl alcohol (3.49%). All of the identified volatile component indicated 99.46% of total AFEOs. The results of the present research were similar by Baldino et al. [29] findings on camphor (14.63%) as one of the main component of AFEOs. Other studies reported that carvacrol was a volatile component of AFEOs [38]. These disagreements maybe caused by climate conditions, soil composition, genetic, stage of maturity, cultivars, plant organs, and extraction conditions, as well as the variations in cultivation [38].

### 3.2. Effect of CH-AFEOs Coating on Proximate Composition and pH

The proximate composition among treatments showed similar values for ash, fat, protein and moisture contents, which indicates that chitosan and AFEOs had no significant (*p* > 0.05) effects on chicken fillets composition (Table 2). The results of present research are in agreement with those observed by Alirezalu et al. [21]. The authors showed that the inclusion of natural antioxidants in ɛ-polylysine, chitosan, and nisin had no significant effects on frankfurter-type sausage proximate composition. Agregán et al. [39] also reported similar results in the chemical composition of pork patties by applying natural antioxidant (macroalgae *Fucus vesiculosus* extract). In the same way, de Carvalho et al. [40] evaluated the impact of guarana (*Paullinia cupana*) seed and pitanga (*Eugenia uniflora* L.) leaf extracts on lamb patties and reported no significant differences in chemical compositions among treatments.

**Figure 1:**
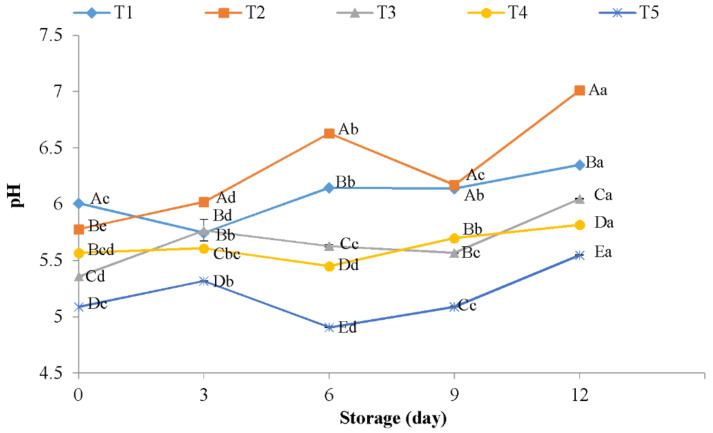
pH values of chicken meat coated with chitosan containing AFEOs during refrigerated storage. T1: Negative control; T2: Distilled water; T3: 1% CH coating + 500 ppm AFEOs; T4: 1% CH coating + 1000 ppm AFEOs; T5: 1% CH coating + 1500 ppm AFEOs. ^a–d^ Mean values during storage not followed by a common letter differ significantly (*p* < 0.05). ^A–E^ Mean values among meat samples not followed by a common letter differ significantly (*p* < 0.05).

On the other hand, pH values in meat and meat products can highly affected microbial balance and function of bacteriostatic, which can lead to a low shelf life. These values are usually under 6 in fresh meat [41]. The changes in pH values of chicken meat between coated treatments during refrigerated storage are showed in Figure 1. As expected, the pH of the chicken fillet samples increased among refrigerated storage. The production of lactic acid bacteria and the accumulation of alkaline components produced by psychrotrophic bacteria and the autolytic activity of the autochthonous enzymes may be the main reason for the change of pH during storage [42,43]. This aforementioned increase was significantly (*p* < 0.05) higher in uncoated samples (negative control and treated with distilled water). At day 12, treated samples with distilled water displayed higher values than those obtained in fillets coated with 1% CH + 1500 ppm AFEOs (7.01 vs. 5.55, respectively). The antibacterial properties of chitosan and AFEOs could be responsible for the lower pH values observed in coated samples. This impact of chitosan films on pH of meat and meat products are in agreement with the results found by other authors in chilled meat [44]. In the same way, Vaithiyanathan et al. [45] and Berizi et al. [46] reported similar behaviour in chicken meat and other food model systems treated with chitosan in combination with natural preservatives.

### 3.3. Effect of CH-AFEOs Coating on TBARS and TVB-N

Shelf life and quality attributes of meat and meat products are highly affected by oxidation reactions, particularly lipid and protein [47]. TBARS are used as an important indicator for the measurement of secondary products of oxidation, especially aldehydes, which resulted from the lipid oxidation of polyunsaturated fatty acids [48]. The effects of chitosan-based coating with AFEOs are displayed in Table 3. TBARS values increased continuously during refrigerated storage, being samples coated with chitosan and AFEOs (T4 and T5) those that displayed significantly (*p* < 0.05) lower values at the end of storage (1.61 and 1.64 vs. 1.92 and 2.10 mg MDA/kg for T5 and T4, vs. negative control and samples treated with distilled water, respectively). Similar results were reported by Liu et al. [44], who evaluated the impact of chitosan films incorporated with natural preservatives on chilled meat. Jonaidi Jafari et al. [49] studied the effect of chitosan coating with ethanolic extract of propolis on the quality of chicken fillets. The authors reported a less increase of TBARS values in treated samples (<0.6 mg MDA/kg in samples coating with chitosan and 2% of ethanolic extract of propolis) compared to those observed in control (>0.8 mg MDA/kg). These lower TBARS values in coated samples may be related to low availableness of oxygen on meat surfaces or chelating impact of chitosan with metal ions [50]. Furthermore, the high antioxidant properties of AFEOs observed by Orhan et al. [28], would also lead to a less increase in TBARS values during storage. Therefore, as expected, chitosan coatings incorporated with AFEOs allowed to extend the shelf life of meat samples by their antioxidative properties. Similar results were observed by Pabast et al. [51] and Fang et al. [52] in lamb meat and fresh pork using chitosan-based coatings with natural antioxidants.

TVB-N value, which mainly includes amines and ammonia, is one of the most important indicators in meat and meat products shelf life [53]. The TVB-N results of chicken samples during refrigerated time are presented in Table 3. In this study, the initial TVB-N values were between 8.7 and 17.9 mg/100 g for treated samples (T5) and samples treated with distilled water (T2), respectively. These values indicate the allowable situation for applied chicken meat. During storage the TVB-N values in all meat samples increased exponentially, with a rate significantly (*p* < 0.05) higher in untreated samples (182.3 vs. 25.3 mg/100 g for T2 and T5, respectively). According to permitted limit of TVB-N values (25 mg/100 g) in meat and meat products, related to loss of freshness and microbiological contamination, control samples (T1 and T2) exceeded this level on day 3. However, treated samples with CH and AFEOs can effectively reduce the production of volatile nitrogen bases under acceptability limits until day 9 (18.3, 19.7, and 18.3 mg/100 g for samples coated with CH and 500, 1000, and 1500 ppm of AFEOs, respectively). The results of the present work are in agreement with those found by Mojaddar Langroodi et al. [54]. The authors showed that CH coating in combination with other natural antioxidants (Sumac extract and *Zataria multiflora* Boiss oil) could significantly reduce TVB-N formation.

In addition, it can be observed that by increasing the EOs concentration, TVB-N values increased more slowly. At day 12, the coated samples containing 1500 ppm AFEOs displayed significantly lower TVB-N values (25.3 vs. 28.2 and 54.3 mg/100 g for samples coated with CH and 500, 1000, and 1500 ppm of AFEOs, respectively). The results of TVB-N values are in paralleled with microbiological results. In fact, the TVB-N results observed among treatments are in agreement with the changes observed in pH, since the antibacterial properties of chitosan and AFEOs could be responsible for the lower pH values in coated samples. Therefore, the lower microbial growth observed in treated samples would lead to lower TVB-N values [49,55].

### 3.4. Effect of CH-AFEOs Coating on TPC

**Figure 2:**
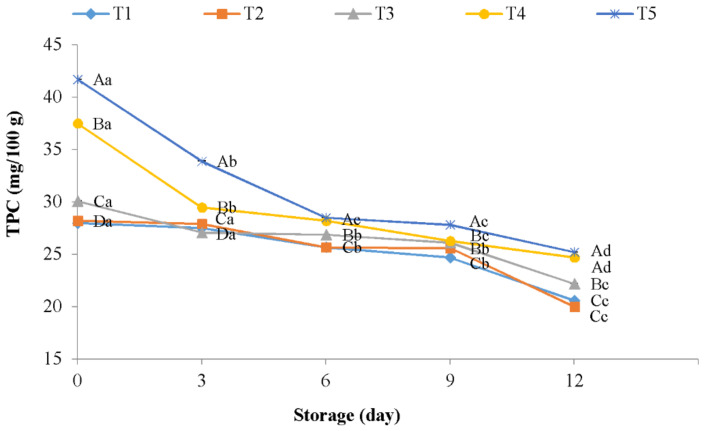
Total phenolic content of chicken meat coated with chitosan containing AFEOs during refrigerated storage. T1: Negative control; T2: Distilled water; T3: 1% CH coating + 500 ppm AFEOs; T4: 1% CH coating + 1000 ppm AFEOs; T5: 1% CH coating + 1500 ppm AFEOs. ^a–d^ Mean values during storage not followed by a common letter differ significantly (*p* < 0.05). ^A–D^ Mean among meat samples not followed by a common letter differ significantly (*p* < 0.05).

Phenolic compounds, which have potential techno-functional, antioxidant, and antimicrobial properties, are highly present in natural sources like plants extracts and EOs [56]. The effects of chitosan coating in combination with AFEOs on phenolic content of chicken meat are shown in Figure 2. At day 0 of storage, phenolic content in chicken samples coated with chitosan and AFEOs ranged from 30.10 to 41.70 mg GA/100 g, whereas the phenolic content in negative control samples was significantly (*p* < 0.05) lower (28.20 mg GA/100 g). The highest phenolic content in treated samples is related to the fact that phenolic compounds are one of the main components of EOs [10]. During the storage period, phenolic compounds in all meat samples decreased significantly (*p* < 0.05). However, treated samples continued to be those that showed the highest contents at day 12, displaying values between 22.20 and 25.20 mg GA/100 g, while negative control and meat treated with distilled water reached to 20 and 20.60 mg GA/100 g, respectively. The decrease in phenolic compounds observed in chicken samples could be attributed to oxidation reactions that take place during storage period [47].

Similar results were found with the use of type of coating materials and natural extracts in meat products. In this regard, Alirezalu et al. [20] evaluated the effects of ɛ-polylysine in combination with natural plant extracts (olive leaves, green tea, and stinging nettle) in frankfurter-type sausage. The authors observed that the samples treated with mixed plant extracts showed significantly higher amounts of phenolic contents compared to control (9.80 vs. 0.07 mg GA/100 g for treated sausages samples and control samples on day 45 of storage, respectively). Similar results with natural plant extracts (rosemary or Chinese mahogany) in fresh chicken sausage were reported by Liu et al. [33].

### 3.5. Effect of CH-AFEOs Coating on Color Parameters

Color is one of the most important parameters in meat and meat products quality, since its stability could compromise the sensory properties of the product and therefore the consumer acceptance [57]. The color indexes (L*: Lightness, a*: Redness and b*: Yellowness) of chicken meat samples were significantly (*p* < 0.05) affected by both coating and refrigerated period (Table 4). L* values of all samples decreased during refrigerated period (Table 4); however, the rate of this reduction was significantly (*p* < 0.05) lower in coated samples. The antioxidant and antimicrobial properties of CH and AFEOs would lead to higher L* in coated samples. At day 12, chicken samples coated with CH + 1500 ppm AFEOs and treated with distilled water showed the highest (36.38) and lowest (25.83) values, respectively. These results are in agreement with those found by Alirezalu et al. [21], who reported a similar trend for lightness in sausages treated with chitosan in combination with other natural antioxidants.

All meat samples revealed a reduction in a* during refrigerated period. The formation of free radicals from lipid oxidation and met-myoglobin may be the main reasons for the reduction of a* values [14,58]. Higher a* values were observed in coated samples compared to those found in negative control, which as mentioned above may be due to the high antioxidant properties of CH and AFEOs. A similar trend in the reduction of a* value in lamb burgers treated with natural extracts was reported by De Carvalho et al. [40].

Regarding yellowness, this parameter is highly affected by the enzymatic browning reactions that occur during the refrigerated storage of meat samples [59]. However, samples coated with CH and high concentration of AFEOs showed significantly (*p* < 0.05) higher b* values than those found by negative control samples at the end of storage (23.66 vs. 20.38 for T5 and T1, respectively).

### 3.6. Effect of CH-AFEOs Coating on Microbiological Analysis

The results of TVC, coliform, molds, and yeasts are shown at Table 5. At day 0, TVC counts in treated samples ranged between 2.27 and 2.33 Log CFU/g, which is significantly lower than those obtained for negative control (4.48 Log CFU/g). These initial bacterial numbers reflect the high antimicrobial properties associated with the use of CH coating and AFEOs in meat samples. Chitosan coating containing AFEOs led to approximately 3 Log CFU/g reduction in TVC from those obtained by control. Increase in the thickness of the chitosan coating not only have inhibitory effects against microbial growth but also could maintain the quality and stability of samples. However, it had been proved that 1% chitosan could also have efficient impacts on meat quality and shelf life. Considering the acceptable limitations of TVC counts (6 Log CFU/g) in fresh poultry meat [60,61], the samples coated with CH in combination with the highest dose of AFEOs displayed acceptable levels at the end of storage time (Table 5), which reflects the possibility of using this coating to extend the shelf life of a highly perishable product, such as fresh chicken meat, ensuring its safety. The results of the present study are in agreement with those found by Jonaidi Jafari et al. [49] on chicken fillets coated with chitosan and ethanolic propolis extract. Bazargani-Gilani et al. [62] also evaluated the effects of chitosan edible coating with plant EOs on chicken breast meat, also reporting the possibility of using the combination of chitosan and EOS to extend the storage time by 10 or 15 days the storage time, which are in agreement with the results found in the present work. Cationic property of chitosan allows to electrostatic interaction between NH3 group (as a positive charges) on of the glucosamine monomer in chitosan molecules and microbial cell membrane (negative charges) led to the leakage of intracellular components could be the reason of antimicrobial properties of chitosan coating, which has been described by Duan et al. [63]. In the other hand, the selective permeability of chitosan [58], which decrease the oxygen transfer to the meat and meat products might be the main reason of extended stability and shelf life.

The meat and meat products surfaces are highly susceptible for molds and yeasts growth, which can lead to spoilage and negative impacts on safety and organoleptic attributes. The chicken meat samples coated with CH + 1500 ppm AFEOs displayed significantly (*p* < 0.05) higher inhibitory effects against molds and yeasts during storage. At the beginning of storage, molds, and yeasts ranged between 1.0 and 3.66 Log CFU/g for samples coated with CH + 1500 ppm AFEOs and distilled water, respectively, which increased significantly (*p* < 0.05) reaching values between 4.27 and 8.02 Log CFU/g at day 12, respectively. In the case of coliforms, a group of microorganisms known as hygienic quality indicators in meat and meat products [64], the counts increased during storage. The rate of this increase was significantly (*p* < 0.05) lower in coated samples with CH + AFEOs (especially in 1500 ppm AFEOs), displaying values after 12 days of storage of 3.87 Log CFU/g compared to values of 8.58 and 8.84 Log CFU/g observed in negative control samples.

To sum up, CH coating + 1500 ppm AFEOs showed the highest antimicrobial activities against TVC, coliforms, molds, and yeasts. The results of present work are in agreement with those reported by Alirezalu et al. [21], who support the use of chitosan (1%) in combination with plant extracts as antimicrobial ingredients in frankfurter-type sausage. Similar results were obtained by Berizi et al. [46] with the combination of chitosan edible coating and pomegranate peel extract.

### 3.7. Effect of CH-AFEOs Coating on Sensory Properties

**Figure 3:**
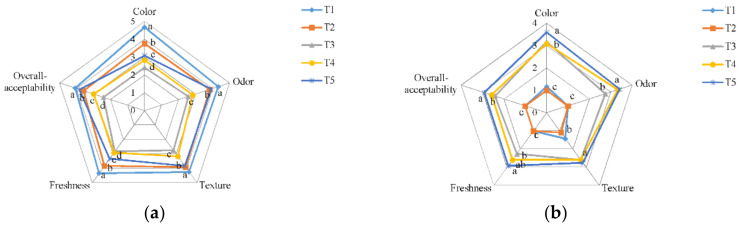
Sensory properties of chicken meat coated with chitosan containing AFEOs at day 0 (**a**) and day 9 (**b**) during storage at 4 °C. T1: Negative control; T2: Distilled water; T3: 1% CH coating + 500 ppm AFEOs; T4: 1% CH coating + 1000 ppm AFEOs; T5: 1% CH coating + 1500 ppm AFEOs. ^a–d^ Mean values among meat samples not followed by a common letter differ significantly (*p* < 0.05).

The effects of CH coating with AFEOs on organoleptic properties of meat samples are illustrated in Figure 3a,b. The results observed on day 0 showed that coated meat samples with CH and AFEOs had a negatively effect on sensory attributes. Despite at the beginning of storage, the highest and lowest sensory scores were for negative control and CH containing 500 ppm AFEOs, and the scores changed as storage progressed since the samples coated with CH and AFEOs displayed significantly (*p* < 0.05) higher scores in all of the attributes evaluated. This could be associated with the higher microbial growth and oxidation reactions that occur in negative control, resulting in a sharply decrease during storage of its sensory properties in comparison with coated samples. Again, the results showed that samples coated with CH + 1500 ppm AFEOs displayed the best results, so this coating could significantly preserve sensory attributes of fresh chicken meat during storage. These results corroborate those previously found by Kanatt et al. [65], who reported that CH coating has no negative effects on organoleptic characteristics of meat and meat products. Furthermore, similar results were previously found by Petrou et al. [66] in chicken breast meat coated with chitosan and oregano oil.

## 4. Conclusions

The results of the current research revealed that chitosan-based coatings with AFEOs allow deceleration of the microbial growth and the undesirable chemical reactions that occur in meat during storage and, therefore, can extend the shelf life of chicken fillets. The presence of natural antioxidant and antimicrobial components in the composition of AFEOs and chitosan are the main responsible for these characteristics. Coated samples remained within acceptable range of quality-chemical factors, such as TBARS, TVB-N, and pH, for longer time. The outcomes of this study showed that coating based chitosan with 1500 ppm AFEOs had the best inhibitory effects on the oxidative activity and microbial growth. The results also revealed that chitosan coating incorporated with 1500 ppm AFEOs can significantly prolong the stability of chicken breast meat and could be suggested as potential coating materials in meat and meat products.

## Figures and Tables

**Table 1:** Essential oil components of *Artemisia fragrance* used for chicken meat treatments.

Name	Area (%) Essential Oil
4-carene	0.42
Methyl Cinnamat	0.25
3-carene	0.20
β-Cymene	1.37
p-Cymene	0.45
Camphene	0.9
Cis-Salvene	0.2
l-Phellandrene	0.47
Sabinene	0.46
α-Terpinolene	0.75
α-Pinene	0.2
β-Phellandrene	0.51
β-Pinene	0.19
γ-Terpinene	0.69
verbenene	0.18
1,8-Cineole	21.04
4-Terpineol	2.65
l-Camphor	11.87
cis-Jasmone	0.54
Isobornyl alcohol	3.49
l-Carvone	1.15
Myrtenal	0.17
Myrtenol	2.16
Pinocarvone	0.25
Piperitone	0.98
Sabinyl acetate	1.63
Thujone	40.21
Sesquiterpenes (ST)	0.37
Germacrene-D	0.38
Copaene	0.36
Oxygentated Sesquiterpenes (OST)	2.08
Carvacrol	1.12
Cis-Davanone	0.94
Others (OTH)	0.40
1-Octen-3-ol	0.46

**Table 2:** Chemical composition (%) of chicken meat coated with chitosan containing AFEOs during storage at 4 °C.

Sample	Moisture	Fat	Ash	Protein
T1	76.51 ± 1.63	1.37 ± 0.14	1.20 ± 0.04	20.90 ± 0.96
T2	76.02 ± 1.79	1.40 ± 0.07	1.10 ± 0.09	20.92 ± 0.99
T3	76.26 ± 1.76	1.41 ± 0.14	1.02 ± 0.02	21.11 ± 0.35
T4	76.15 ± 0.42	1.39 ± 0.23	1.10 ± 0.03	21.34 ± 0.83
T5	76.89 ± 1.44	1.41 ± 0.09	1.14 ± 0.04	20.51 ± 0.85

The results were expressed as mean values ± standard deviation. T1: Negative control; T2: Distilled water; T3: 1% chitosan (CH) coating + 500 ppm AFEOs; T4: 1% CH coating + 1000 ppm AFEOs; T5: 1% CH coating + 1500 ppm AFEOs. There were no significant differences among treatments.

**Table 3:** Changes in thiobarbituric acid reactive substances (TBARS) and total volatile base nitrogen (TVB-N) values of chicken meat coated with chitosan containing AFEOs during storage at 4 °C.

	Sample	Storage (Day)				
		0	3	6	9	12
TBARS (mg MDA/kg)	T1	1.03 ± 0.006 ^Ae^	1.16 ± 0.011 ^Bd^	1.45 ± 0.017 ^Bc^	1.81 ± 0.014 ^Ab^	1.92 ± 0.020 ^Ba^
	T2	1.03 ± 0.004 ^Ae^	1.64 ± 0.017 ^Ad^	1.73 ± 0.026 ^Ac^	1.87 ± 0.011 ^Ab^	2.10 ± 0.007 ^Aa^
	T3	1.01 ± 0.003 ^Ad^	1.10 ± 0.002 ^Bd^	1.33 ± 0.029 ^Cc^	1.62 ± 0.023 ^Bb^	1.75 ± 0.023 ^Ca^
	T4	0.98 ± 0.017 ^Ad^	1.09 ± 0.001 ^Bd^	1.28 ± 0.017 ^Cc^	1.47 ± 0.026 ^Cb^	1.64 ± 0.029 ^Da^
	T5	0.96 ± 0.008 ^Ae^	1.06 ± 0.006 ^Bd^	1.21 ± 0.018 ^Dc^	1.41 ± 0.018 ^Cb^	1.61 ± 0.020 ^Da^
TVB-N (mg/100 g)	T1	15.6 ± 0.001 ^Bd^	36.5 ± 0.012 ^Bc^	37.2 ± 0.008 ^Bc^	105.0 ± 0.004 ^Bb^	151.2 ± 0.004 ^Ba^
	T2	17.9 ± 0.021 ^Ad^	39.4 ± 0.003 ^Ac^	41.9 ± 0.008 ^Ac^	178.3 ± 0.008 ^Ab^	182.3 ± 0.008 ^Aa^
	T3	10.0 ± 0.005 ^Cd^	14.1 ± 0.004 ^Cc^	17.1 ± 0.012 ^Cb^	18.3 ± 0.004 ^Db^	54.3 ± 0.004 ^Ca^
	T4	10.0 ± 0.004 ^Ce^	12.8 ± 0.004 ^Dd^	14.1 ± 0.004 ^Dc^	19.7 ± 0.004 ^Cb^	28.2 ± 0.004 ^Da^
	T5	8.7 ± 0.009 ^Dd^	11.1 ± 0.004 ^Ec^	11.4 ± 0.006 ^Ec^	18.3 ± 0.004 ^Db^	25.3 ± 0.004 ^Ea^

T1: Negative control; T2: Distilled water; T3: 1% CH coating + 500 ppm AFEOs; T4: 1% CH coating + 1000 ppm AFEOs; T5: 1% CH coating + 1500 ppm AFEOs. ^a–e^ Mean values in the same row not followed by a common letter differ significantly (*p* < 0.05). ^A–E^ Mean values in the same column not followed by a common letter differ significantly (*p* < 0.05).

**Table 4:** Color indexes of chicken meat coated with chitosan containing AFEOs during storage at the 4 °C.

	Sample	Storage (Day)				
		0	3	6	9	12
L*	T1	30.94 ± 0.618 ^Ca^	26.94 ± 0.529 ^Bb^	27.77 ± 0.309 ^Bb^	27.44 ± 0.277 ^Cb^	27.00 ± 0.600 ^Bb^
	T2	31.11 ± 0.493 ^Ca^	33.00 ± 0.003 ^Aa^	29.16 ± 0.254 ^Ba^	33.22 ± 0.309 ^Ba^	25.83 ± 0.984 ^Bb^
	T3	38.61 ± 0.111 ^Ba^	34.33 ± 0.346 ^Ab^	37.11 ± 0.829 ^Aa^	33.72 ± 0.364 ^ABb^	37.05 ± 0.242 ^Aa^
	T4	38.83 ± 0.346 ^Aa^	35.50 ± 0.010 ^Aab^	36.77 ± 0.547 ^Aa^	32.11 ± 0.618 ^Bb^	38.05 ± 0.388 ^Aa^
	T5	37.93 ± 0.693 ^Aa^	34.44 ± 0.433 ^Ab^	30.44 ± 0.454 ^Bc^	36.59 ± 0.746 ^Aa^	36.38 ± 0.484 ^Aa^
a*	T1	0.66 ± 0.166 ^ABb^	4.94 ± 0.444 ^Aa^	−0.22 ± 0.400 ^Aab^	−2.00 ± 0.384 ^Ab^	−5.33 ± 0.509 ^Ac^
	T2	−1.33 ± 0.254 ^BCb^	2.88 ± 0.200 ^Aa^	−3.77 ± 0.388 ^Bc^	−3.83 ± 0.166 ^Cc^	−4.22 ± 0.337 ^Cd^
	T3	−3.50 ± 0.096 ^Ca^	−3.33 ± 0.096 ^Ca^	−3.94 ± 0.293 ^Bb^	−4.72 ± 0.242 ^Dc^	−4.77 ± 0.400 ^Bc^
	T4	−2.38 ± 0.995 ^Ca^	−4.11 ± 0.364 ^Cb^	−4.94 ± 0.474 ^Cd^	−2.27 ± 0.293 ^Ba^	−4.83 ± 0.096 ^Bc^
	T5	1.46 ± 0.062 ^Aa^	−1.88 ± 0.493 ^Bc^	−0.77 ± 0.146 ^Ab^	−1.68 ± 0.168 ^Ac^	−4.33 ± 0.509 ^Cd^
b*	T1	19.11 ± 0.963 ^Cbc^	18.94 ± 0.146 ^Cc^	17.94 ± 0.111 ^Cd^	16.11 ± 0.200 ^Ce^	20.38 ± 0.146 ^Ca^
	T2	17.83 ± 0.146 ^Dd^	20.00 ± 0.192 ^Cb^	17.87 ± 0.055 ^Cd^	18.16 ± 0.192 ^Bc^	22.22 ± 0.585 ^Ba^
	T3	17.16 ± 0.254 ^Dd^	22.83 ± 0.166 ^Ab^	21.61 ± 0.493 ^ABc^	21.11 ± 0.055 ^Ac^	24.83 ± 0.333 ^Aa^
	T4	20.83 ± 0.192 ^BCb^	21.61 ± 0.111 ^ABa^	22.11 ± 0.200 ^Aa^	20.33 ± 0.192 ^Ab^	22.22 ± 0.222 ^Ba^
	T5	23.33 ± 0.461 ^Aa^	20.27 ± 0.293 ^Bb^	20.44 ± 0.146 ^Bb^	19.26 ± 0.156 ^ABb^	23.66 ± 0.288 ^ABa^

T1: Negative control; T2: Distilled water; T3: 1% CH coating + 500 ppm AFEOs; T4: 1% CH coating + 1000 ppm AFEOs; T5: 1% CH coating + 1500 ppm AFEOs. ^a–e^ Mean values in the same row not followed by a common letter differ significantly (*p* < 0.05). ^A–D^ Mean values in the same column not followed by a common letter differ significantly (*p* < 0.05).

**Table 5:** Evaluation of microbiological counts (Log CFU/g) in chicken meat coated with chitosan containing AFEOs during storage at 4 °C.

	Sample	Storage (Day)				
		0	3	6	9	12
TVC	T1	4.48 ± 0.012 ^Ac^	5.89 ± 0.196 ^Bb^	7.77 ± 0.004 ^Aa^	7.95 ± 0.001 ^Aa^	8.01 ± 0.012 ^Aa^
	T2	4.47 ± 0.115 ^Ac^	6.22 ± 0.114 ^Ab^	8.04 ± 0.022 ^Aa^	8.13 ± 0.020 ^Aa^	8.21 ± 0.015 ^Aa^
	T3	2.29 ± 0.013 ^Be^	3.02 ± 0.026 ^Cd^	4.42 ± 0.018 ^Bc^	5.67 ± 0.005 ^Bb^	7.41 ± 0.012 ^Ba^
	T4	2.33 ± 0.032 ^Be^	2.85 ± 0.009 ^Cd^	3.45 ± 0.017 ^Cc^	5.55 ± 0.008 ^Bb^	6.90 ± 0.027 ^Ca^
	T5	2.27 ± 0.015 ^Bd^	2.30 ± 0.007 ^Dd^	3.27 ± 0.013 ^Cc^	4.61 ± 0.010 ^Cb^	5.32 ± 0.014 ^Da^
Coliforms	T1	4.21 ± 0.132 ^Ad^	4.71 ± 0.052 ^Bc^	7.82 ± 0.009 ^Ab^	8.05 ± 0.014 ^Ab^	8.58 ± 0.019 ^Aa^
	T2	4.08 ± 0.044 ^Ad^	6.18 ± 0.006 ^Ac^	8.23 ± 0.012 ^Ab^	8.38 ± 0.005 ^Ab^	8.84 ± 0.004 ^Aa^
	T3	1.47 ± 0.013 ^Bc^	1.31 ± 0.318 ^Dc^	3.33 ± 0.017 ^Bb^	3.64 ± 0.028 ^Bb^	4.47 ± 0.004 ^Ba^
	T4	ND	1.78 ± 0.015 ^Cd^	3.12 ± 0.067 ^BCc^	3.50 ± 0.046 ^Bb^	4.18 ± 0.009 ^BCa^
	T5	ND	1.47 ± 0.012 ^Dd^	2.86 ± 0.019 ^Cc^	3.03 ± 0.009 ^Cb^	3.87 ± 0.031 ^Ca^
Molds and yeast	T1	3.34 ± 0.022 ^Be^	3.68 ± 0.046 ^Bd^	6.03 ± 0.004 ^Bc^	7.28 ± 0.011 ^Bb^	7.55 ± 0.015 ^Ba^
	T2	3.66 ± 0.029 ^Ae^	4.13 ± 0.025 ^Ad^	6.96 ± 0.006 ^Ac^	7.78 ± 0.012 ^Ab^	8.02 ± 0.020 ^Aa^
	T3	1.60 ± 0.012 ^Ce^	1.94 ± 0.013 ^Cd^	3.97 ± 0.023 ^Cc^	4.59 ± 0.007 ^Cb^	4.92 ± 0.026 ^Ca^
	T4	1.30 ± 0.013 ^De^	1.84 ± 0.012 ^Cd^	3.49 ± 0.057 ^Dc^	4.38 ± 0.046 ^Db^	4.61 ± 0.013 ^Da^
	T5	1.00 ± 0.015 ^Ee^	1.69 ± 0.015 ^Dd^	2.99 ± 0.025 ^Ec^	3.97 ± 0.022 ^Eb^	4.27 ± 0.015 ^Ea^

T1: Negative control; T2: Distilled water; T3: 1% CH coating + 500 ppm AFEOs; T4: 1% CH coating + 1000 ppm AFEOs; T5: 1% CH coating + 1500 ppm AFEOs. ND: Not detected. ^a–e^ Mean values in the same row not followed by a common letter differ significantly (*p* < 0.05). ^A–E^ Mean values in the same column not followed by a common letter differ significantly (*p* < 0.05).

## Data Availability

The data presented in this study are available on request from the corresponding author.
